# The Living and Dead Hands

**Published:** 1915-10-02

**Authors:** 


					THE LIVING AND DEAD HANDS.
METROPOLITAN HOSPITALS, 1904 to 1913.
A.?Table Showing the Sources of Income oe the
Metropolitan Hospitals and Dispensaries in the Ten
Years 1904-1913. See The Hospital, June 12, 1915
Year
1904
1905
1906
1907
1908
1909
1910
1911
1912
1913
Prom the Living
J2 ?n\
d .*?
g.2 ?
O "? "S I
'-3 Oi '
D? ?
?r d o
^ g g a-
_o o 5 in ,
3^?c
O Oo
603,425
610,130
635,377
653,320
712,461*
701,458
703,841
738,622
741,439
845,908*
50
49
58
48
56
57
50
53
55
52
Prom the Dead
2 3
5 S"
a a*
v -3 Eh
Jd-S
?go?
<p Q*C<1
03 _,
d g d
Hgg
? a
o*?
258,567
264,790
272,704
296,218
290,491
294,206
309,320
329,253
338,425
349,295
22
21
25
22
23
24
22
24
25
22
"$3
xn ^"oj
.2-2 H
O'OiH
d d o
2 a
O 3
O o
w o
319,303
373,914
186,284
407,918
265,493
240,523
394,478
322,949
272,090
424,339
28
30
17
30
21
19
28
23
20
26
"3 2
o ?
EH a
1,181,295
1,248,834
1,094,365
1,357,456
1,268,445
1,236,187
1,407,639
1,390,824
1,351,954
1,619,542
I 1 1 II II
.* Includes ?72,565 and ?103,970, the amounts received
by the London Hospital in 1908 and 1913, respectively, as
the result of its quinquennial appeals in these years.
LIVERPOOL HOSPITALS, 1907 to 1913-
B.?Table Showing the Sources op Income of the
Liverpool Hospitals for the Seven Years 1907-1913.
Tear
1907
1908
1909
1910
1911
1912
1913
j^rom the Living
1
"S ?i ^
a ? o Si;
Its ? %?
'S m d a o
5 ? 2 2-<
;o0ftrt
*3 "t? ^ ^
r5 c3 ca ^ n
? Q P. ?,
58,096
59,209
55,837
69,377
66,631
70,233
5129,954
a o
gH
77
75
62
73
59
68
78
From the Dead
2 3
iS f 3
a
o> .3 H
!i=
>? CO
? s
? 3
o-s
14,038
14,138
14,940
15,582
15,606
16,543
17,955
19
18
17
16
14
16
11
-ES 'J
<3
2 d
?s'-sS
<s a o
?o ?
<u e,??
'-'So
fcJ S
O 3
Bo
O
2,785
5,467
18,261
10,739
30,467
16,815
17,976
4
7
21
11
27
16
11
"3 3
74,919
78,814
89,038
95,698
112,704
103,591
165,885
I 1 I I I 1 1
[Note.?The fluctuations in column 1 are due largely to
periodical as well as annual appeals on account of extra
ordinary expenditure on buildings, etc.]

				

